# Norovirus GII.P16/GII.2–Associated Gastroenteritis, China, 2016

**DOI:** 10.3201/eid2307.170034

**Published:** 2017-07

**Authors:** Yuanyun Ao, Jinjin Wang, Hua Ling, Yaqing He, Xiaogeng Dong, Xuan Wang, Jingyao Peng, Hailong Zhang, Miao Jin, Zhaojun Duan

**Affiliations:** Chinese Center for Disease Control and Prevention, Beijing, China (Y. Ao, M. Jin, Z. Duan);; Shanghai Ocean University, Shanghai, China (J. Wang);; Chongqing Center for Disease Control and Prevention, Chongqing, China (H. Ling, J. Peng);; Shenzhen Center for Disease Control and Prevention, Shenzhen, China (Y. He, H. Zhang);; Fengtai District Center for Disease Control and Prevention, Beijing (X. Dong);; Nanjing Center for Disease Control and Prevention, Nanjing, China (X. Wang)

**Keywords:** gastroenteritis, norovirus, recombinant, GII.P16/GII.2, viruses, China, enteric infections

## Abstract

During October–December 2016, the number of norovirus outbreaks in China increased sharply from the same period during the previous 4 years. We identified a recombinant norovirus strain, GII.P16-GII.2, as the cause of 44 (79%) of the 56 outbreaks, signaling that this strain could replace the predominant GII.4 viruses.

Noroviruses, the main cause of nonbacterial acute gastroenteritis outbreaks (*1*), are positive-sense, single-stranded RNA viruses within the family *Caliciviridae* (*2*). The genome contains 3 open reading frames (ORFs). ORF1 encodes nonstructural proteins, including an RNA-dependent RNA polymerase (RdRp), ORF2 encodes a capsid protein (viral protein 1 [VP1]), and ORF3 encodes a minor capsid protein (VP2). On the basis of RdRp and VP1 gene sequences, noroviruses are classified into 7 genogroups (GI–GVII) (*3*). GI, GII, and GIV noroviruses can infect humans; GI and GII viruses, the most common, include at least 31 distinct genotypes (*3*).

Since 2002, GII.4 viruses have been the most common norovirus genotype circulating worldwide (*3**,**4*). However, during the winter of 2014–15 in parts of Asia, a new GII.17 strain emerged as the major cause of acute gastroenteritis outbreaks (*5*–*7*), suggesting that non–GII.4 norovirus might become the predominant genotype. We report that, in late 2016 in China, the number of norovirus outbreaks increased significantly over the same period during the previous 4 years (56 in 2016 vs. 6 in 2013, 11 in 2013, 36 in 2014, and 14 in 2015). A GII.P16-GII.2 virus caused 79% of the 56 outbreaks in 2016.

## The Study

Acute gastroenteritis outbreaks, defined as >20 patients with vomiting and diarrhea associated with a common source of infection within 1 week, are reported to the National Emergent Public Health Event Information Management System established by the Chinese Center for Disease Control and Prevention (China CDC). Fecal specimens from each outbreak were tested for noroviruses by the local Center for Disease Control and Prevention using commercial real-time reverse transcription PCR (RT-PCR) kits (BioPerfectus Technology Co., Jiangsu, China; Aodong Inspection & Testing Technology Co., Shenzhen, China). At least 3 norovirus-positive samples from each outbreak were transported to the China CDC for study. The China CDC Institutional Review Board for human subject protection approved this study.

**Figure 1:**
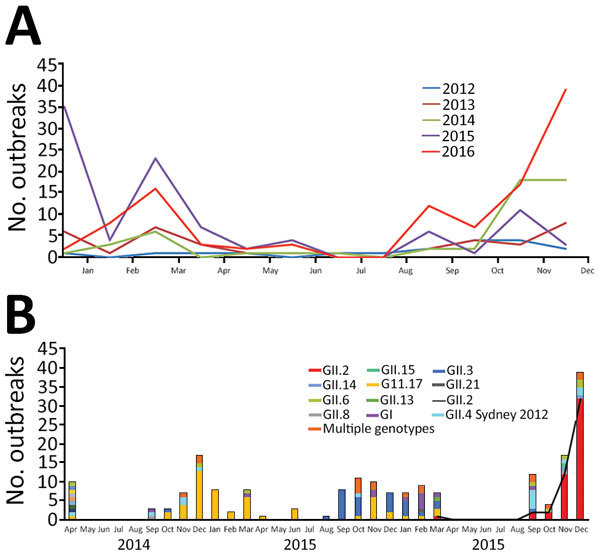
Norovirus outbreaks, China. A) Outbreaks reported to the China Centers for Disease Control and Prevention during 2012–2016. B) Genotype (capsid) distribution of norovirus outbreaks during April 2014—December 2016.

During 2012–2016, a total of 313 norovirus outbreaks in 24 provinces and 96 cities in China were reported to the National Emergent Public Health Event Information Management System; 109 (35%) were reported in 2016, mostly in winter. In November and December 2016, norovirus outbreaks increased sharply, accounting for 56 (51%) of the 109 norovirus outbreaks (Figure 1, panel A). These outbreaks occurred mainly in kindergartens (48%) and schools (39%) (Technical Appendix Table 1).

Partial capsid genes were amplified by conventional RT-PCR for genotyping, as described previously (*8*). Of the outbreaks,25 (78%) of 32 were caused by GII.17 noroviruses during the winter of 2014–15 and 9 (60%) of 15 by GII.3 noroviruses during the winter of 2015–16. In 2016, samples from 94 (86%) of the 109 outbreaks were genotyped. The most common norovirus genotype was GII.2 (49 [52%]), followed by GII.3 and multiple genotypes (8 [9%] each); GII.4Sydney 2012 (7 [7%]); GII.17 and GII.6 (5 [5%] each); and others (12 [13%]). Of the 8 outbreaks involving multiple genotypes, 3 included GII.P16-GII.2 (Figure 1, panel B). Of the 49 GII.2 outbreaks in 2016, 44 (90%) were reported during November and December 2016. Thirty-eight (78%) GII.2 norovirus outbreaks occurred in kindergartens. Samples from all GII.2 norovirus outbreaks were selected for dual genotyping using conventional RT-PCR spanning the ORF1/ORF2 region, as developed by the US Centers for Disease Control and Prevention (Atlanta, GA, USA) (J. Vinjé, pers. comm., 2017 Mar 1). Genotyping results showed 48 outbreaks were caused by GII.P16/GII.2 norovirus, and 1 outbreak was caused by the GII.P2/GII.2 norovirus. The first GII.P16/GII.2 norovirus was detected in August 2016.

We obtained complete capsid sequences and nearly complete RdRp sequences from samples from 30 GII.2 outbreaks using nested PCR (Technical Appendix Table 2). Furthermore, 2 complete genomes (JS1208 and BJSMQ) were determined, showing the highest nucleotide identities (95%) to those of the HS255/2011/USA (GenBank accession no. KJ407074.2) and Miyagi1/2012/JP (GenBank accession no. LC145787.1) strains. Overall, 35 GII.2 VP1 sequences were 99% nt identical to each other, and the 28 RdRp sequences were closely related to the GII.P16 norovirus recently detected in Germany (*9*). All nucleotide sequences obtained were deposited in GenBank (accession nos. KY421121–KY421185).

The predicted 35 GII.2 VP1 amino acid sequences we determined were aligned with those for GII.2 strains from the 1970s to 2016 that are available in GenBank, and <15 aa mutations were found in the VP1 region, suggesting that the GII.2 norovirus is still circulating stably worldwide. This finding is consistent with a recent report showing that the non–GII.4 norovirus remains static (*10*). The x-ray crystal structure of the capsid-protruding domain of GII.2 strain Snow Mountain virus (SMV) (GenBank accession no. KF429769) was recently determined (*11*). Compared with the histo-blood group antigen (HBGA) binding surface of strain SMV, the GII.2 strains in this study had 15 aa mutations in VP1, including 8 aa mutations at residues 335–349 around the HBGA binding site I in the P2 domain, although the conserved set of residues (Asn352, Arg353, Asp382, and Gly445) required for binding the fucose moiety of HBGAs in SMV was unchanged. Sequence analysis of the RdRp region revealed that 28 GII.2 strains in this study differed from GII.P16/GII.4 strain CA3477 by ≈1–5 aa mutations. A sequence analysis of 35 predicted GII.2 VP2 sequences showed mutations (5–10 divergent residues) at the C-terminus of VP2 compared with those of HS255 and Miyagi1. ORF1 of JS1208 and BJSMQ also differed from that of GII.P16/GII.4 strain CA3477 by 11 aa.

**Figure 2:**
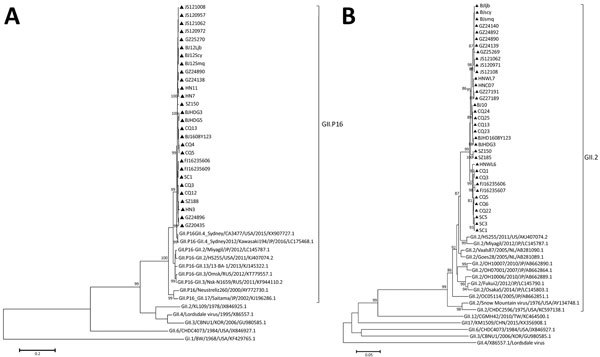
Phylogenetic analyses of the newly identified GII.2 noroviruses in China, reconstructed based on RNA-dependent RNA polymerase (A) and open reading frame 2 (B) with a representative norovirus using the neighbor-joining method with datasets of 1,000 replicates in MEGA 6.0 software (http://www.megasoftware.net). Triangles indicates the positions of the GII.2 norovirus newly identified in 8 cities from 7 provinces. Scale bars indicate nucleotide substitutions per site.

Phylogenetic analysis using VP1 showed that 35 strains in this study formed a subcluster independent of the GII.2 subcluster; the strains were most closely related to the norovirus GII.2 subcluster that included HS255 and Miyagi1. However, the RdRp of 28 GII.2 strains formed a single cluster and showed maximum relatedness to those of GII.P16/GII.4 strain CA3477 (Figure 2). The complete genomes of GII.P2/GII.2 strain Malaysia (GenBank accession no. JX846925.1) and GII.P16/GII.4 strain CA3477 (GenBank accession no. KX907727.1) were used as query sequences to predict possible recombination breakpoints of the JS1208 genome by SimPlot software version 3.5.1 (http://sray.med.som.jhmi.edu/SCRoftware/simplot). The recombination breakpoint was predicted to be at nucleotide position 5088 at the boundary between ORF1 and ORF2.

## Conclusions

In China, the number of norovirus outbreaks increased substantially in late 2016, greatly surpassing norovirus activity reported during the same months of the previous 4 years. Most of these outbreaks were associated with a GII.P16-GII.2 strain, similar to a recently reported pattern in Germany (*9*). Seventy-eight percent of the GII.2 outbreaks occurred in kindergartens. The exact reason is unknown but is in line with the model presented by Parra et al. (*10*), in which non-GII.4 genotypes seem to infect infants more frequently because adults have built immunity to different genotypes over time. This finding should be confirmed by serum inhibition/neutralization tests for specific genotypes or strains, such as GII.P16-GII.2, in the population. Continuous surveillance is needed to explore the epidemiologic, clinical, and evolutionary characteristics of this GII.P16-GII.2 strain. Additional studies should explore the mechanisms behind the emergence of this active strain.

Technical Appendix Setting distribution of 109 norovirus outbreaks in China, 2016; primers used to amplify the complete genome, open reading frames 2–3, and RNA-dependent RNA polymerase of GII.P16/GII.2 noroviruses.
