# Supplementary material for: Norovirus GII.P16/GII.2–Associated Gastroenteritis, China, 2016
Source: Emerg Infect Dis. 2017 Jul;23(7):1172–5. doi: 10.3201/eid2307.170034 (PMC5512504; doi:10.3201/eid2307.170034)
Supplement: Technical Appendix — Setting distribution of 109 norovirus outbreaks in China, 2016; primers used to amplify the complete genome, open reading frames 2–3, and RNA-dependent RNA polymerase of GII.P16/GII.2 noroviruses. [file 17-0034-Techapp-s1.pdf]

Article DOI: https://dx.doi.org/10.3201/eid2307.170034

Norovirus GII.P16/GII.2–Associated Gastroenteritis Outbreaks, China, 2016

Technical Appendix

Technical Appendix Table 1. Settingss of 109 norovirus outbreaks, China, 2016

Setting	All genotypes, no. (%)	GII.2, no. (%)
Kindergarten	52 (48)	38 (78)
School	44 (39)	7 (14)
University	3 (3)	1 (2)
Community	3 (3)	1 (2)
Hospital	2 (2)	2 (4)
Workplace	5 (5)	0

Technical Appendix Table 2. Primers used to amplify the complete genome, open reading frames 2–3, and RNA-dependent RNA polymerase of GII.P16/GII.2 noroviruses

Primer name	Sequence (5′→3′)	Position
NVORF1–1F*	ATGAAGATGGCGTCTAACGAC	1–21
NVORF1–1R1*	CTGTGAGAGTGGATGCCAC	1562–1544
NVORF1–1R2*	CTGCTATTCGAGCCAGCAGG	1418–1399
NVORF1–2F*	CAATGAGCTAGCTATGGTGA	1220–1201
NVORF1–2R1*	GAACTCGTCATCTTCTGACT	2599–2580
NVORF1–2R2*	GACAAACGCAGCGCCGGCA	2485–2467
NVORF1–3F*	TGGTTCCACGTATGTCCTTGA	2290–2310
NVORF1–3R1*	ACCGAGGTAGGCAGGCTCA	3712–3694
NVORF1–3R2*	GTGTGGCACAGATGACGGTG	3542–3523
NVORF1–4F*	CGCATGGTTGGCGGACAGATG	3362–3382
NVORF1–4R1*	GCAGTGCCATGAGCTGTATG	4901–4882
NVORF1–4R2*	AGTCCAGTACATCTGCCTC	4825–4807
COG2F (sense)†	CARGARBCNATGTTYAGRTGGATGAG	5003–5028
G2SKF (sense)†	CNTGGGAGG GCGATCGCA A	5046–5064
VNT20†	GAGTGA CCGCGGCCGCT$_{20}$	
RdRp1500F1*	GTGCACACTGCAGCAGCC	3494–3511
RdRp1500F2*	CACCGTCATCTGTGCCACAC	3523–3542
RdRp1500R*	CGTCATTCGACGCCATCTTCAT	5106–5085

*Primers designed in this study.

†Primers from Katayama K, Shirato-Horikoshi H, Kojima S, Kageyama T, Oka T, Hoshino F, et al. Phylogenetic analysis of the complete genome of 18 Norwalk-like viruses. Virology. 2002;299:225–39.
